# Efficacy of an imidacloprid/flumethrin collar against fleas and ticks on cats

**DOI:** 10.1186/1756-3305-5-82

**Published:** 2012-04-27

**Authors:** Dorothee Stanneck, Eva M Kruedewagen, Josephus J Fourie, Ivan G Horak, Wendell Davis, Klemens J Krieger

**Affiliations:** 1 Bayer HealthCare AG, Animal Health Division, D-51368 Leverkusen, Germany; 2 ClinVet International, P.O. Box 11186, Universitas, Bloemfontein 9321, South Africa; 3 Department of Zoology and Entomology, University of the Free State, Bloemfontein 9301, South Africa; 4 Department of Veterinary Tropical Diseases, Faculty of Veterinary Science, University of Pretoria, Onderstepoort 0110, South Africa; 5 Bayer HealthCare LLC, Animal Health, 12809 Shawnee Mission Parkway, Shawnee KS 66216, USA

**Keywords:** Imidacloprid, Flumethrin, Collars, Efficacy, Safety, Fleas, Ticks, Cats

## Abstract

**Background:**

The objectives of the studies listed here were to ascertain the therapeutic and sustained efficacy of 10% imidacloprid (w/w) and 4.5% flumethrin (w/w) incorporated in a slow-release matrix collar, against laboratory-infestations of fleas and ticks on cats. Efficacy was evaluated against the flea *Ctenocephalides felis felis*, and the ticks *Ixodes ricinus*, *Amblyomma americanum* and *Rhipicephalus turanicus*. The number of studies was so large that only a general overview can be presented in this abstract.

**Methods:**

Preventive efficacy was evaluated by infesting groups of cats (n = 8-10) with *C. felis felis* and/or *I. ricinus, A. americanum* or *R. turanicus* at monthly intervals at least, for a period of up to 8 months. Efficacy against fleas was evaluated 24 to 48 h after treatment and 24 h after infestation, and against ticks at 6 h (repellent) or 48 h (acaricidal) after infestation. Efficacy against flea larvae was evaluated over a period of 8 months by incubating viable flea eggs on blanket samples after cat contact. In all cases efficacy was calculated by comparison with untreated negative control groups.

**Results:**

Efficacy against fleas (24 h) generally exceeded 95% until study termination. *In vitro* efficacy against flea larvae exceeded 92% until Day 90 and then declined to 67% at the conclusion of the study on Day 230.

Sustained acaricidal (48 h) efficacy over a period of eight months was consistently 100% against *I. ricinus* from Day 2 after treatment, 100% against *A. americanum*, except for 98.5% and 97.7% at two time-points, and between 94% and 100% against *R. turanicus*.

From Day 2 until 8 months after treatment the repellent (6 h), efficacy was consistently 100% against *I. ricinus,* and between 54.8% and 85.4% against *R. turanicus*.

**Conclusion:**

The rapid insecticidal and acaricidal properties of the medicated collars against newly- acquired infestations of fleas and ticks and their sustained high levels of preventive efficacy have been clearly demonstrated. Taking into account the seasonality of fleas and ticks, the collars have the potential to prevent the transmission of vector-borne diseases and other conditions directly associated with infestation throughout the season of parasite abundance.

## Background

Fleas and ticks are amongst the most well known groups of ectoparasites on domestic cats and dogs, and have probably coexisted with humans and their domestic animals for millenia. It can readily be assumed that from the earliest times they were detested by humans because of the irritation associated with their bites or attachment on their own bodies, and in later times because of the distress they caused to their pets. However, their roles as vectors of a wide range of viral, bacterial and protozoan diseases and also of helminths have only relatively recently been recognized [1-3]. As a consequence their biology, vectorial capacity and control have become a priority in veterinary and medical research. However, control is not as straightforward as simply applying ectoparasiticides to a host animal. Especially as a large part of a flea’s life cycle, and the major portion of a tick’s life cycle is spent off the host [4,5]. Furthermore, the various free-living stages of both fleas and ticks may survive for several months before finding a host.

### Fleas

Fleas are probably amongst the most important ectoparasites of domestic cats. They may be present on cats that are confined to apartments, or to family homes with outside yards or gardens, or on animals that are practically free-ranging in barns and stables on farms. The cat flea, *Ctenocephalides felis felis* is responsible for most of the problems associated with flea infestation on these animals. The ancestors of fleas of the genus *Ctenocephalides* appear to have originated in Africa [6], and over time *C. felis felis* and to a lesser degree *C. canis* have adapted to feeding on domestic cats. *C. felis felis* has now been transported worldwide by humans on their pets, while its sibling species, *Ctenocephalides felis strongylus* has remained a parasite of small wild felids in Africa [7].

The three larval stages of cat and dog fleas as well as the pupa are free-living within the resting and loafing environment of cats, while the adults are for all practical purposes permanent parasites on their hosts [4]. Under conditions of optimal temperature and humidity, the life cycle from egg-laying until the emergence of adult fleas from pupae, can be completed in 23 days [4].

In addition to the irritation caused by fleas, cats often develop a condition known as flea allergy dermatitis [8] resulting from repeated exposure to flea bites. This condition is characterised by pruritus, leading to licking, nibbling and scratching that can result in alopecia, skin damage and other signs of self-trauma. Furthermore *C. felis felis* is an intermediate host of the tapeworm *Dipylidium caninum *[1].

Various studies aimed at determining the species composition of the fleas infesting cats, and in some cases also their seasonal abundance, have been conducted. In northern Greece, Hungary, Austria and south-western England cats were infested with both *C. felis felis* and *C. canis*. Infestation rates with *C. felis felis* varied between 93.7 and 97.4 %, while infestation with *C. canis* was significantly lower and varied between 3.7 and 6.2 % [9-12]. In Tirana, Albania flea-infested cats examined in late autumn harboured only *C. felis felis *[13], and in Florida, USA, 185 of 200 feral cats examined during June to September were infested with *C. felis felis*, while nine cats also harboured *Pulex irritans* and 11 were infested with *Echidnophaga gallinacea *[14]. In South Africa, Horak *et al*. [15] collected eight flea species from five wild felid species and only one species from seven feral cats. The most numerous fleas on these animals were *Ctenocephalides damarensis* followed by *E. gallinacea*, and while *C. felis strongylus* was present on three caracals, no *C. felis felis* was recovered.

### Ticks

Unlike the kennel tick, *Rhipicephalus sanguineus* which well-nigh exclusively infests domestic dogs, no tick species in any country is a specific parasite of cats to the exclusion of all, or nearly all, other host species. There are, however, a number of tick species that, in addition to infesting domestic cats infest domestic dogs as well as wild felids and several other animals [16,17]. Ticks are sensitive to external climatic conditions, and with notable exceptions most species are confined to particular regions of the world and few species have successfully colonized other regions [18]. *Rhipicephalus turanicus* is one of the species that is present in several regions, and it infests cats as well as a variety of other host species [18].

There are four stages in the life cycle of ixodid or hard ticks, namely eggs, larvae, nymphs and adults [5]. Once the eggs have hatched, all subsequent stages require a blood meal before they can continue their development [5]. The larvae, nymphs and adults each feed for a few days to a week, representing a total feeding period of approximately three weeks. The whole life cycle frequently takes a year to complete, although for some species it may take two or more years to complete.

All three stages of some tick species may by preference infest the same host species [18], thus making on-host control of all stages possible. The immature stages of other tick species may infest one host species and the adults another. Thus, if the larvae and nymphs infest rodents and the adults infest cats, control of the immature stages is nearly impossible. Furthermore, all stages, and more particularly the adults of some species, may infest a large variety of domestic and wild animals, and although they can be controlled on the domestic animals, fresh infestations will always be possible from wildlife in the vicinity.

A number of surveys on the tick species that infest cats have been conducted. For instance, in Austria and Great Britain *Ixodes ricinus* and *Ixodes hexagonus* were common parasites of cats [11,16,19], thus indicating that the latter tick is also an important parasite of these animals when they are allowed free access to the outdoors. In Florida, USA, Akucewich *et al*. [14] found that only five of 200 feral cats examined were infested with ticks and that these five cats harboured a total of only nine ticks of which two were adult *Amblyomma americanum*, five *Dermacentor variabilis*, and single specimens of *Ixodes scapularis* and *Rhipicephalus sanguineus*. In contrast Horak & Matthee [20] and Horak *et al*. [17] collected 2, 225 ixodid ticks belonging to 16 species from 150 tick-infested domestic cats examined in surveys in South Africa. Depending on the locality, six of these species, including *R. turanicus*, are regular parasites of cats and wild felids, with the remainder considered to be ‘stragglers’.

### Active ingredients

Imidacloprid belongs to the group of chloronicotinyl compounds and has excellent insecticidal properties, coupled with low mammalian toxicity. In certain formulations it also has a long-lasting residual efficacy, and has been registered in the USA and EU member states as a veterinary medicinal product for use on dogs and cats since 1996/7. Flumethrin belongs to the group of α-cyano-pyrethroids, and although it has some insecticidal properties, it is particularly effective as an acaricide for the control of ticks, and has been registered as such in EU member states for use on companion and food-producing animals since 1986.

Imidacloprid and flumethrin have now been combined in a slow-release matrix collar formulation containing 10% imidacloprid (w/w) and 4.5% flumethrin (w/w). The imidacloprid component of the collar is aimed at the treatment and control of fleas, and the flumethrin component at the treatment and prevention of infestation by ticks. The medicated collar is intended for the long-term protection of cats exposed to infestation by fleas and ticks.

The objectives of the present studies were to demonstrate the efficacy of the medicated collars against existing populations of fleas and ticks as well as their sustained effectiveness over a period of several months in preventing re-infestation.

## Methods

All studies were conducted under the respective national animal protection legislation framework, and ethical approval was therefore obtained in all cases before study start.

Unless otherwise stated, each study was designed to determine the therapeutic and sustained efficacy of collars containing imidacloprid 10.5 % (w/w) and flumethrin 4.5 % (w/w) against laboratory-bred strains of the cat flea, *C. felis felis* or against the ticks *I. ricinus*, *A. americanum* and *R. turanicus* on artificially infested cats. In each study the parasite burdens of a group of untreated control cats were compared with those of a group of cats fitted with the medicated collars. Most cats were infested on multiple occasions at pre-determined intervals and were then examined for live parasites after each re-infestation.

The animals included in the various studies were mostly short-haired domestic cats weighing between 2.5 and 7 kg. They had also not been treated with an acaricide, or insecticide, or a compound with an insect-growth regulating activity during the previous 60 to 90 days. All cats were maintained and handled with due regard for their welfare, and were acclimatized to the cat holding facilities approximately 7 to 21 days prior to the commencement of a study. Cats were housed in pens in indoor animal units that conformed to the national standards for floor area, lighting and temperature of the various countries in which the studies were conducted. Water was available *ad libitum* and an adequate amount of a commercial cat food was provided daily towards their maintenance.

Three colonies of parent stock fleas were used for infestation of the cats. One colony was used for studies conducted in Germany, another for the study conducted in the USA and a third for the studies carried out in South Africa. These colonies had been maintained for at least two years. The fleas in the colonies were reared on domestic cats and their eggs were collected and incubated with flea rearing medium (dried, powdered beef blood, ground dog food, yeast and sand). The ensuing adult fleas were collected and the numbers stipulated in the protocols for the various studies counted and placed in separate containers. The fleas placed in the containers were unfed and less than 14 days old. The containers were opened on the cats’ necks and the fleas were allowed to disperse in their fur.

Parent stock ticks were reared on artificially infested rabbits, dogs or sheep.

The *I. ricinus* used in studies conducted in Germany were obtained from two sources, namely a colony maintained by Dr Dautel in Berlin and from the Bayer Animal Health colony maintained at Monheim. A laboratory-bred strain of *Rhipicephalus turanicus* originating from a tick collected from a domestic cat at Onderstepoort was used for the study against this tick species in South Africa. The ticks are routinely fed on rabbits. Unfed adult *A. americanum* for the study conducted in the USA were provided by Oklahoma State University, Stillwater. The colony was started in 1976 with ticks from the wild, and fresh, engorged females from the natural population are introduced every two years. The ticks are maintained on sheep and rabbits.

The ticks that were used for infestation in the various studies, were unfed, and at least one week old. Cats were lightly sedated or restrained by hand and pre-determined numbers of ticks were released onto their backs or necks and allowed sufficient time to disperse into the fur before the animals were released.

Approximately four days before the collars were fitted all cats in a study were infested with a prescribed number of fleas or ticks of the species to be targeted. One or two days after this infestation flea or tick counts were conducted by combing the cats’ fur for fleas or hand-searching their pelage for ticks. The cats were ranked in descending order on their pre-treatment parasite counts, and animal ID’s were used to break ties. Thereafter they were randomly allocated to treatment groups. Each treated and each untreated control group of cats consisted of at least seven individuals.

Flea counts were performed by using a fine-toothed flea comb (the teeth were 1mm apart and approximately 10 mm long) to recover fleas present in the animal’s fur. Several strokes of the comb were made over the head and ears, over the neck, lateral areas, dorsal strip from shoulder blades to base of tail, tail and anal area, fore legs and shoulders, hind legs, abdominal area from chest to inside hind legs and feet, each time moving in the same direction following the lie of the hair coat. Fleas showing no movement were considered dead. Fleas were removed from the teeth of the comb or collected from the sheet on which the cat was combed and transferred to plastic bags or other containers for counting at a later date.

Tick counts were performed by intensive examination and palpation of the whole skin surface of each cat. During *in situ* counting or upon removal, ticks were specified as to whether they were dead or alive. Following removal, live and dead ticks from each cat were categorized according to their attachment and engorgement status (Table 1). Engorgement was evaluated in non-evident cases by squeezing the tick over filter paper in order to detect traces of blood. During the tick counting procedure male and female *A. americanum* and *R. turanicus* were counted, whereas only female *I. ricinus* were counted as specified in the respective EMA guideline (below), because the males of *Ixodes* species seldom attach.

**Table 1:** Classification of ticks on their state of attachment and engorgement and whether they were alive or dead

**Category**	**Condition**	**Attachment status**
1	Live	Unattached
2	Live	Attached, unengorged*
3	Live	Attached, semi-engorged to engorged**
4	Dead	Unattached
5	Dead	Attached, unengorged*
6	Dead	Attached, semi-engorged to engorged**

* No filling of the idiosoma evident.

** Conspicuous filling of the idiosoma.

One or two days before the collars were fitted all animals in a particular study were infested with the stipulated number of fleas, or of ticks, or with both fleas and ticks of the species to be targeted. On Day 0 the collars were fitted to the necks of the cats allocated to the treatment group. The collars that were used were designed for animals weighing less than 8 kg, and weighed 12.6 gm and were 35 cm long. After the collar had been secured by means of the buckle, the loose end was passed through the retaining loop. The excess collar was cut off approximately 2 cm from the retaining loop. At pre-determined time intervals on the day that the collars were fitted all animals were carefully observed for adverse signs that could be ascribed to the collars or to the active ingredients which they contained.

Two days after collars had been fitted to cats that had been infested with fleas and/or ticks, flea and/or tick counts were carried out. At predetermined intervals thereafter all the cats in some of the studies were re-infested with fleas or ticks or with both depending on the target species. A day after each re-infestation flea counts were carried out and two days after re-infestation tick counts were performed. This procedure was repeated at approximately 28-day intervals for a total period of more-or-less 35 weeks after the collars had been fitted.

The experimental design of the various studies is schematically summarized in Table 2.

**Table 2:** Schematic Experimental Design of studies aimed at testing the efficacy of an imidacloprid 10.5%/flumethrin 4.5% collar against ectoparasites on cats

**Study day (week)**	**Activity**
−10 (approximately)	Acclimatization to housing
−7	Pre-study flea or tick infestations
−4	Select cats with highest flea or tick counts, and allocate them to treatment groups consisting of at least 7 untreated cats and 7 treated cats
−2, -1	Flea and/or tick infestation
0	Fit collars to all cats in treatment group
2	Flea and tick counts
7 (=Week 1)	Flea and/or tick infestation
8, 9	Flea counts Day 8, tick counts Day 9
28 (=Week 4)	Flea and/or tick infestation
29, 30	Flea counts Day 29, tick counts Day 30
56 (=Week 8)	Flea and/or tick infestation
57, 58	Flea counts Day 57, tick counts Day 58
84 (=Week 12)	Flea and/or tick infestation
85, 86	Flea counts Day 85, tick counts Day 86
112 (=Week 16)	Flea and/or tick infestation
113, 114	Flea counts Day113, tick counts Day 114
140 (=Week 20)	Flea and/or tick infestation
141, 142	Flea counts Day 142, tick counts Day 142
168 (=Week 24)	Flea and/or tick infestation
169, 170	Flea counts Day 169, tick counts Day 170
196 (=Week 28)	Flea and/or tick infestation
197, 198	Flea counts Day 197, tick counts Day 198
224 (=Week 32)	Flea and/or tick infestation
225, 226	Flea counts Day 225, tick counts Day 226
238 (=Week 34)	Flea and/or tick infestation
239, 240	Flea counts Day 239, tick counts Day 240
245 (=Week 35)	Flea and/or tick infestation
246, 247	Flea counts Day 246, tick counts Day 247

All efficacy values have been based on a comparison of transformed geometric means of the individual parasite counts of treated and of untreated control animals. Geometric means better reflect the non-normal distribution of the numbers of parasites recorded on individual animals within the small groups of cats of necessity included in a laboratory study.

Efficacy has been determined as described in document EMEA/CVMP/EWP/005/2000-Rev.2 of the European Medicines Agency.

Efficacy against fleas was calculated as follows:

(1) $$\text{Efficacy}\;\left(\%\right)=100\times \left(\text{mc}–\text{mt}\right)/\text{mc},\text{where}$$

mc = geometric mean number of live fleas on the untreated control group of cats. mt = geometric mean number of live fleas on the treated groups of cats.

The ticks collected from each cat were sorted into six categories according to their engorgement status and whether they were alive or dead (Table 1). Ticks showing no movement were considered dead.

Acaricidal efficacy was calculated as follows:

(2) $$\text{Acaricidal efficacy}\left(\%\right)=100\times \left(\text{Gmc}–\text{Gmt}\right)/\text{Gmc}$$

Where Gmc = Geometric mean number of live ticks (categories 1–3) on cats in the untreated control group at a specific time point. Gmt = Geometric mean number of live ticks (categories 1–3) on cats in the treated group at a specific time point.

Sustained acaricidal efficacy against subsequent re-infestations, was calculated using the same formula as above, but in this instance Gmt = Geometric mean number of live and dead ticks (categories 1–3 & 6). According to the EMEA guidelines, dead engorged ticks (Category 6) must be included with the 3 categories of live ticks as these ticks had succeeded in engorging before being killed.

The various studies were conducted at facilities in Germany, North America and in South Africa and the experimental design of each one is not necessarily identical. For the sake of uniformity, efficacy determinations have thus been allocated to 28-day or 4-week blocks or windows, and any determination of efficacy during a 4-week window has been included in the window within which it had been calculated.

### Fleas

#### Efficacy against adult fleas

Cats in five long-term studies spanning 34 weeks, were infested with *C. felis felis*, fitted with collars, and had flea counts done as summarized in the Schematic Experimental Design (Table 2), while speed of efficacy was determined as described in the

#### Onset of efficacy and speed of kill

The onset of efficacy study was aimed at determining the efficacy of the collars against fleas that were applied at the same time as the collars were fitted. In another study the speed at which fleas were killed after infestation (repellent effect) was determined. Both are described in the following.

In the first study collars were fitted to the cats in the treated group and immediately thereafter all the cats were infested with fleas and efficacy assessed 24 h later. In the second study, collars were fitted to the cats in the treated group on Day 0 and all the cats were infested with fleas late in the afternoon on Day 6 and fleas were collected 12 h later on Day 7. Once these collections had been completed the same two groups of cats were infested on Day 7 and fleas collected 6 h later. Finally the same groups of cats were again infested with fleas on Day 7 and fleas collected 2 h thereafter. Efficacy could thus be determined at 2, 6 and 12 h after infestation.

#### In vitro larvicidal efficacy

In the study described here the effect of residues of imidacloprid and flumethrin on blankets used by collared cats on the development of flea larvae was measured. Fleecy polyester blankets were clamped onto thick cardboard squares which were then placed on the floor of transport boxes. The 20 cats in the study were confined in these boxes for 4½ h on Day-9 and again on Day −8 before the collars were fitted. After the collars had been fitted to the cats in the treated group, all the cats in both groups were confined in the boxes for 4½ h on Days 13 and 14 and thereafter for 4½ h on two consecutive days prior to each efficacy assessment day. After each 9 h period of exposure (4½ h + 4½ h) of the blankets to the cats, the blankets were removed from the boards and a circular sample, 8.5 cm in diameter, was cut from the centre of each blanket. The cardboard squares were discarded and fresh squares and fleecy blankets prepared for each subsequent period of exposure. The blanket samples were placed in Petri dishes and frozen at approximately −20°C for at least 24 h. After removal from the freezer the Petri dishes were allowed to reach room temperature. Approximately 50 one-day old flea eggs were placed in the middle of each blanket-sample and about 0.5 g of flea-rearing medium (dried, powdered beef blood, ground dog food, yeast and sand) was distributed in a very thin layer over the surface of each sample. The samples were incubated at 25 to 28°C and 70 ± 5% relative humidity for 28 days. To facilitate counting of the adult fleas that emerged, the Petri dishes were frozen at −20°C for at least 2 h. The geometric mean numbers of fleas on the blanket samples exposed to the untreated control cats compared to those on the blanket samples exposed to collared cats were used to calculate efficacy.

#### Flea allergy dermatitis (FAD)

In addition to other regular observations and clinical examinations, the cats in all the long-term studies involving repeated infestation with *C. felis felis*, were monitored for signs of flea allergy dermatitis. The numbers of animals exhibiting signs of this condition were recorded and palliative treatment, free from any insecticidal side effects, was immediately initiated.

#### Results and discussion

##### Efficacy against adult fleas

The results of the studies involving adult *C. felis felis* are summarized in Table 3.

**Table 3:** **Efficacy of imidacloprid 10%/flumethrin 4.5% collars 24 hours after each infestation of cats with** *** Ctenocephalides felis felis***

**Study day or week**	**Activity and efficacy (%)**				
Day - 1	Each cat infested with approximately 100 fleas				No infestation
Day 0	Collars fitted to cats in treated groups				
Day 2 (48h)	100*	100	100	100	100 fleas applied
Day 3	-	-	-	-	90.7
Day 7	98.6	100	-		100**
Day 20	-	-	100*	100	-
Week 4	99.5	100	99.9	-	99.6
Week 8	96.8	100	100	100	97.6
Week 12	98.6	99.8	99.9	99.7	97.5
Week 16	97.9	99.6	99.0	100	100
Week 20	99.3	97.7	99.5	100	100
Week 24	97.7	97.2	96.5	97.9	98.4
Week 28	96.1	92.1	97.3	99.2	96.9
Week 32	96.1*	94.5	96.5	98.7	-
Week 34	96.6	89.1	97.2	91.5	95.5

* Efficacy determined 48 h after infestation.

** Infestation took place on Day 9.

The cats in these studies were each infested with100 fleas on numerous occasions and the mean flea burdens of the untreated control cats varied between 18.3 and 79.8, thus representing a robust level of infestation throughout the study periods. Therapeutic efficacy against one-day old infestations of *C. felis felis* measured two days after the collars had been fitted in four of the long-term studies was 100 %. Sustained efficacy measured 24 h after regular re-infestation remained above 96% for 34 weeks in two of the studies, and exceeded 97% until Week 32 in another. In the fourth study sustained efficacy exceeded 95% until Week 24 and exceeded 90% until Week 32. In the fifth study sustained efficacy exceeded 95% from Week 4 to Week 34.

In one of the studies a transitory unexpectedly low efficacy was recorded at 14 days and in a second study at 20 weeks after the collars had been applied. These lower efficacies could be related directly to deviations from the study protocols. In the first study Elizabethan collars had been fitted to the cats to prevent them from removing ticks and fleas by grooming, but when it became obvious that these collars were interfering with the natural process of grooming they were removed. In the second study a large number of fleas apparently escaped during the infestation process at Week 20. All the fleas resulting from the faulty infestations were removed by thorough combing, and in both cases the cats were re-infested within a week and efficacies above 99% were recorded. These re-infestations and their results could thus be included within the applicable 4 week windows of efficacy (Table 3).

The reason for the decrease in efficacy of the collars recorded after 24 weeks in one of the studies could be due the usual shedding of hair by cats in autumn (flea infestation took place in mid October). This would have led to a more rapid loss of active ingredient on the shed hair than could be replenished from the collar. This phenomenon cannot always be avoided in such long-term studies, but on the other hand it emphasises the advantage of a low dose, long-term, continuous release matrix device over a single spot-on treatment during autumn. In the case of a spot-on treatment the animal would have to be re-treated after an intensive period of hair shedding.

In various studies on cats in which the sustained efficacy of single applications of spot-on formulations of imidacloprid were tested against re-infestation with *C. felis felis*, efficacy exceeded 95% for at least four weeks after treatment, declining fairly rapidly thereafter [21-23]. The efficacy of the imidacloprid/flumethrin medicated collars against re-infestation with *C. felis felis* exceeded 95% for at least 24 weeks after the collars had been fitted, and in some cases remained at the 95% level until 34 weeks after treatment. This prolonged period of efficacy exempts the forgetful pet owner from the often onerous task of repeated spot-on treatments for the duration of the flea season.

##### Onset of efficacy and speed of kill

In the study in which infestation took place immediately after the collars had been fitted, 99.8 % of fleas were killed within 24 h (Table 4). In the other study in which the cats had been fitted with collars seven days earlier, all fleas were killed within 2, 6 and 12 h (Table 4).

**Table 4:** Onset of efficacy and speed of kill of imidacloprid 10%/flumethrin 4.5% collars against * Ctenocephalides felis felis* on cats

**Study day**	**Infestation**	**Efficacy (%)**
Day 0	Collars then 100 fleas	
Day 1		99.8*
Day 6	100	
Day 7 (12 h later)		100**
Day 7	100	
Day 7 (6 h later)		100**
Day 7	100	
Day 7 (2 h later)		100**

* Onset of efficacy.

** Speed of kill or repellent effect.

The rapidity with which imidacloprid eliminates fleas on cats after re-infestation has previously been demonstrated with a spot-on formulation [21]. In that study efficacy against *C. felis felis* exceeded 58% within 2 h of re-infestation and 90% within 4 h of re-infestation on Days 7 and 14 after topical treatment. In the present study the collar formulation of imidacloprid and flumethrin was 100% effective against *C. felis felis* within 2 h of re-infestation seven days after the collars had been fitted (Table 4). The enhanced efficacy of the collars compared to that of the spot-on treatment can probably be ascribed to a greater concentration of imidacloprid on the hair of cats wearing collars than that on the hair of cats treated with the spot-on formulation. It is also possible that the strain of fleas used in the present study was more susceptible to imidacloprid than the strain tested in the spot-on treatment. In addition to the imidacloprid component of the collars, the flumethrin component also has insecticidal properties.

##### In vitro larvicidal efficacy

This study endeavoured to simulate the dispersion of the active ingredients of the collar in a cat’s immediate surroundings such as its bedding and regular resting places and the effect of these residues on flea larvae within this environment. The results are presented in Table 5.

**Table 5:** Effect of imidacloprid and flumethrin residues against larvae of *Ctenocephalides felis felis*on blankets that had been in contact with cats wearing medicated collars

**Study day or week**	**Activity and efficacy (%)**	
	**50 flea eggs incubated on 10 control and on 10 in-contact blankets**	
	**Efficacy (%)**	**Mean No. of fleas on control blankets/ No. on in-contact blankets**
Day −8	0	16.7/6.7
Day 0	Collars fitted to treated cats	
Week 2	100	26.5/0
Week 5	100	16.8/0
Week 10	96	32.5/1.3
Week 13	92.9	21.2/1.5
Week 17	73.7	26.2/6.9
Week 21	81.3	26.7/5
Week 25	67.3	26.6/8.7
Week 29	69.9	24.3/7.3
Week 33	67.5	15.4/5

During the first 5 weeks after the collars had been applied no fleas were encountered on blanket samples that had been in contact with the collared group of cats. Thereafter flea numbers on the treated blankets were reduced by 96% at Week 10 and by 92.9% at Week 13 compared to the numbers counted on blankets exposed to the untreated control cats. Reduction in numbers thereafter varied between 67.3% and 81.3%.

The reason for the decrease in efficacy of the imidacloprid/flumethrin residues on the blankets after 90 days (13 weeks) is probably a result of the natural heavy grooming activities of cats. While grooming they are likely to remove the active ingredient on their hair-tips, which is the main source of imidacloprid transferred to the blankets. Initially the decrease in active ingredient is rapidly replenished from the collar; but after 3 months the release of active ingredient becomes slightly slower and the effect of grooming more visible.

The contact of 9 h that treated cats had with blankets during each three or four-week window of the study is equivalent to a single night’s sleep on each occasion. However, even this limited spell of exposure was sufficient to prevent more than 92% of larvae from developing into adult fleas for a period of three months. Efficacy can be expected to be higher and last for longer in a domestic environment, in which a treated cat may spend several hours daily on its bedding or in its favourite resting places. Application of the collar will thus not only kill an existing flea population, but will rapidly also kill fleas in any subsequent infestation (Table 4), and at the same time prevent the vast majority of flea larvae in the cat’s immediate surroundings from developing into adults. This attribute negates the necessity of an additional environmental treatment.

##### Flea allergy dermatitis (FAD)

The numbers of incidents of FAD in the untreated control groups of cats infested with fleas during the various long-term studies, in which the efficacy of the collars was tested, compared to those in the collared groups of cats, are summarized in Table 6.

**Table 6:** Flea allergy dermatitis (FAD) in repeatedly infested untreated control cats compared to similarly infested cats wearing imidacloprid 10%/flumethrin 4.5% collars

**Study period in days**	**Control groups**		**Observations**	**Treated groups**	
	**No. of animals**	**No. with FAD**		**No. of animals**	**No. with FAD**
239	8	1	Moderate FAD	14	0
246	7	3	Severe FAD on Days 114 and 142	14	0
226	8	0	No reaction	24	0
239	10	7	Confirmed FAD throughout study	9	0
239	10	(9)	9 controls mild signs of FAD 1 treated cat, intensive licking	10	(1)
240	10	0	No reaction	10	0
246	8	(1)	Excessive hair loss	8	0
**Total**	**61**	**11 (10)**		**89**	**(1)**

(n) = Number of cats with signs suggestive of FAD.

During the long-term laboratory efficacy studies cats were periodically, but only temporarily exposed to large numbers of fleas, and this was followed by subsequent 4-week periods of non-exposure (Table 3). A number of untreated control animals that had not exhibited FAD at the commencement of the studies, reacted to re-infestation towards the end of the different study periods. Thus a total of 11 cats in three of the seven studies displayed clear signs of FAD, and 10 cats in two of the four remaining studies displayed signs that were suggestive of this condition. Despite repeated re-infestations only a single cat amongst the 89 treated animals showed signs suggestive of FAD and this animal developed alopecia because of intensive licking.

Imidacloprid containing products formulated for topical application have been used for several years as an integral part of the FAD treatment strategy [8,24]. These products are not only highly effective but also act very rapidly. In a large scale field trial conducted at 423 veterinary clinics in Italy, Genchi *et al*. [8] recorded a reduction of more than 90% in the number of cats that were infested with fleas during the four weeks following a single treatment with a topical formulation of imidacloprid (Advantage®). At the same time the percentage of cats exhibiting signs of FAD was reduced from more than 23% to below 6%. Cats exhibiting signs of FAD at eight veterinary clinics in Ontario, Canada, were treated with a topical formulation of imidacloprid (Advantage®) at 28-day intervals [24]. During the 84 days of the study, scores for flea infestation decreased by 89% at 14 days after the first treatment and by 100% at Day 84. At the commencement of the study all the cats presented with pruritus, but this had declined to 12% of the total at the end of the study while the proportion of cats with signs of alopecia decreased from 36% to 12.5%.

The effect of imidacloprid on fleas is extremely rapid and in the present study the imidacloprid/flumetrin collar was 100% effective in killing fleas within 2 h of artificial infestation (Table 4). Thus fleas are likely to be eliminated shortly after getting onto a collared cat and the chances of them feeding and provoking FAD by the secretion of their saliva is likely to be reduced. Despite the excellent efficacy of imidacloprid applied as a topical formulation, treatments at monthly intervals are nevertheless necessary to control new infestations of fleas and prevent FAD. The situation is very different in the case of the medicated collar. Because of the slow, long-term release of imidacloprid from the collar matrix, treated animals are protected for eight months against infestation by fleas. This negates the possibility of a gap in treatment occurring during the flea season in which FAD could be provoked by fresh infestation. Taken in conjunction with the larvicidal effect of imidacloprid on the bedding and other resting places of collared cats the flea challenge that may occur is likely to be minimal in any event.

#### Ticks

##### Methods

###### Therapeutic and sustained efficacy

The studies presented here describe the therapeutic and sustained efficacy of 10% imidacloprid/4.5% flumethrin collars against laboratory infestations of three tick species that infest cats in the field. In these studies infestation with ticks, application of the collars, determination of tick numbers and the assessment of efficacy follow the procedures described in the Methods and summarized in the Schematic Experimental Design (Table 2). The ticks targeted in these studies were *I. ricinus*, *A. americanum* and *R. turanicus* (Tables 7 and 8).

**Table 7:** Efficacy of imidacloprid 10%/flumethrin 4.5% collars 48 hours after each infestation of cats with *Ixodes ricinus*

**Study day or week**	**Activity and efficacy (%)**		
Tick species	*Ixodes ricinus*		
Day −2	20♂, 20♀ ticks		
Day −1	-	20♂, 20♀ ticks	20♂, 20♀ ticks
Day 0	Collars fitted to cats in treated groups		
Day 2 (therapeutic efficacy)	62.8	95.5	54.1
Day 7	100	100	100
Week 4	100	100	100
Week 8	100	100	100
Week 12	100	100	100
Week 16	100	100	100
Week 20	100	100	100
Week 24	100	100	100
Week 28	100	100	*
Week 32	100	100	100
Week 34	100	100	100

* No efficacy determined because of a shortage of ticks.

**Table 8:** Efficacy of imidacloprid 10%/flumethrin 4.5% collars 48 hours after each infestation of cats with *Amblyomma americanum* or *R. turanicus*

**Study day or week**	**Activity and efficacy (%)**	
Tick species	*Amblyomma americanum*	*Rhipicephalus turanicus*
Day - 1	50 ticks (mixed sexes)	50 ticks (25♂ 25♀)
Day 0	Collars fitted to cats in treated group	
Day 2 (therapeutic efficacy)	93.3	88.2
Day 5	100	-
Day 14	100	-
Day 18	-	99.5
Week 4	-	98.1
Week 6	100	-
Week 8	-	94.4
Week 10	100	-
Week 12	-	95.7
Week 14	98.5	-
Week 16	-	94.1
Week 18	97.7	-
Week 22	100	-
Week 24	-	97.8
Week 26	100	-
Week 28	-	95.7
Week 30	100	-
Week 32	-	94.4
Week 34	100	100

###### Onset of efficacy and speed of kill

A short-term study involving eight treated and eight untreated cats was conducted in order to determine the onset of efficacy of the imidacloprid/flumethin collars against infestation with *I. ricinus.* Collars were fitted to the treated cats in this study on Day 0 and directly thereafter all cats were infested with *I. ricinus*. Ticks were removed and counted 48 h later and on the same day the cats were re-infested with *I. ricinus* and efficacy determined 6 h later.

###### Repellent efficacy

Two studies with *I. ricinus* and one with *R. turanicus* were carried out in order to determine the repellent effect of the collars on these tick species. Cats that had been fitted with medicated collars on Day 0 were infested with 20 male and 20 female *I. ricinus* or with 25 male and 25 female *R. turanicus* four weeks later and at 4-weekly intervals thereafter. Six hours after each infestation the ticks on these animals were counted *in situ* and not removed and repellent efficacy determined. Other than this infestation with ticks, application of collars, and assessment of efficacy followed the procedures described in the Methods and summarized in the Schematic Experimental Design (Table 2). However, 48 h after each infestation the ticks were removed and counted. This was done because the repellency studies were designed in such a manner that not only repellent effectiveness could be measured 6 h after each infestation, but also long-term sustained acaricidal efficacy could be determined 48 h after the same infestations (Tables 7, 8).

#### Results and discussion

##### Therapeutic and sustained efficacy

The results of three long-term studies evaluating the therapeutic and sustained acaricidal efficacy of the medicated collars against *I. ricinus* are summarized in Table 7. The cats in these studies were each infested with 20 male and 20 female *I. ricinus* on numerous occasions and the mean burdens of the untreated control cats varied between 3.7 and 14.5 ticks. In view of the fact that only female ticks were counted, these levels of infestation were entirely adequate for the determination of efficacy.

Therapeutic efficacy against one-day old *I. ricinus* was 54.1%, and against two-day old ticks it was 62.8% and 95.5%. Compared to the rather variable therapeutic efficacy, the sustained preventive efficacy of the collars against re-infestation with *I. ricinus* was 100% for the full 34-week duration of the studies. This finding is of particular importance to the owners of cats in Europe, Great Britain and Ireland where a large proportion of cats may be infested with *I. ricinus *[11,16,19].

The variability in therapeutic efficacy can probably be ascribed to the fact that pyrethroids, including flumethrin, generally seem to have a limited degree of efficacy against partially engorged ticks, whose metabolism and body surface have changed dramatically from that of unfed ticks. However, two days after the collars had been fitted the concentration of flumethrin on the haircoat was sufficient to result in a repellent efficacy of 100 % 6 h after infestation (Table 9).

**Table 9:** Repellent acaricidal efficacy of 10%/flumethrin 4.5% collars 6 hours after each infestation of cats with *Ixodes ricinus* or *Rhipicephalus turanicus*

**Study day or week**	**Activity and efficacy (%)**		
Tick species	*Ixodes ricinus*		*Rhipicephalus turanicus*
No. of ticks	20♂, 20♀ ticks	20♂, 20♀ ticks	25♂, 25♀ ticks
Day 0	Collars fitted to cats in treated groups		
Week 4	100	100	83.2
Week 8	100	100	70.8
Week 12	100	100	57.5
Week 16	100	100	54.8
Week 20	100	100	*
Week 24	100	100	85.4
Week 28	100	100	65.7
Week 32	100	100	59.8
Week 34x	100	100	61.1

The results of the single efficacy study against the American lone star tick, *A. americanum* are summarized in Table 8. Taking into account that the cats were infested with 50 ticks at each occasion, mean burdens on the untreated cats were low, ranging from 5.4 to 12.3 ticks. However, because the collars were highly effective this had little if any effect on the outcome.

Two days after the collars were fitted therapeutic efficacy against a one-day old infestation of *A. americanum* was 93.3%, and with the exception of Weeks 14 and 18, when efficacy was 98.5% and 97.7%, preventive efficacy from Day 5 until the conclusion of the study at Week 34 was 100%. The 8-month long sustained high level of efficacy of the collars against *A. americanum* has important implications for pet owners in the USA where this tick infests a variety of hosts including cats and dogs.

Although therapeutic efficacy measured against *R. turanicus* that had been attached for one day was 88.2%, sustained preventive efficacy exceeded 94% for the 8 month duration of the study (Table 8). The cats in this study were infested with 50 ticks on numerous occasions and the mean numbers of *R. turanicus* on the untreated control cats declined erratically, but progressively from 10.1 on Day 2 and 13.4 on Day 18, to 0.7 by week 34. This decline, and particularly because it was progressive, almost certainly indicates that the cats had developed resistance to re-infestation with *R. turanicus*.

##### Onset of efficacy and speed of kill

The medicated collars eliminated 95.5% of *I. ricinus* that had been applied immediately after the collars had been fitted. The collars also killed 100% of *I. ricinus* within 6 h of re-infestation two days after they had been fitted (Table 10). Thus efficacy was already in place within 24 h of simultaneous collaring and tick infestation and repellency (6 h) was already active 2 days after the collars had been fitted. Interpolating these results to conditions in the field implies that most *I. ricinus* climbing onto a cat on the same day that the collars are fitted would be killed, and most if not all *I. ricinus* getting onto cats on which the collars had been in place for two days would be killed or prevented from attaching within 6 h. From the pet owner’s point of view he or she could be confident that their cats would be protected against ticks practically immediately after the animals had been fitted with collars.

**Table 10:** Onset of efficacy and speed of kill of imidacloprid 10%/flumethrin 4.5% collars against *Ixodes ricinus* on cats

**Study day**	**Activity and efficacy (%)**
**Day 0**	Collars then immediately 35 *I. ricinus* (15♂ 20♀)
**Day 2**	95.5
**Day 2**	35 *I. ricinus* (15♂ 20♀)
**Day 2** (6h after re-infestation)	100

* Cats infested with fleas and ticks on the same day that the collars were fitted.

##### Repellent efficacy

In both studies involving *I. ricinus*, the repellent efficacy of the medicated collars was 100% at Week 4 after infestation, and remained at this level for the full 34 week duration of the studies (Table 9). On the other hand repellent efficacy against *R. turanicus* measured 6 h after infestation was very variable, dropping to below 60% on three occasions and never exceeding 90% (Table 9). However, sustained efficacy against the same populations of ticks measured 42 h later exceeded 94% for the duration of the study (Table 8).

The perception of repellent efficacy against ticks can either be based on a true repellent effect in which the ticks do not even attempt to attach to a host. Or it can be based on an extremely rapidly acting killing effect, eliminating any ticks that do climb on to a host within a very short period of time. The EMEA efficacy guidelines for testing ectoparasiticides on cats and dogs allows for a 24 h time interval between infestation and effect in order to claim a repellent effect. In the two studies involving *I. ricinus*, no ticks, dead or alive, were detected on the treated cats 6 h after infestation, thus indicating either an extremely rapid killing effect or a true repellent effect.

### Discussion

A sound knowledge of the seasonal abundance of fleas and ticks is a prerequisite towards determining the optimum time at which to commence with treatment. Unfortunately it would seem as if few long-term surveys, during which ectoparasites have been collected at regular intervals from a particular group of cats or from a single cat, have been done. Most studies appear to rely on results obtained from cats presented at veterinary practices throughout the year or during a particular season or seasons. However, several long-term studies have been done on dogs. Consequently the seasonality of ectoparasites on domestic pets discussed here will rely on results obtained from cats and from dogs. Some seasonal data obtained from wild felids, on which the tick species encountered are also regular parasites of domestic cats, will also be included.

In general terms, infestation with fleas in both the Northern and Southern Hemispheres commences in spring or increases from a low base during spring to reach peak numbers in mid or late summer followed by a decline towards winter [10,14,15,25,26]. It is thus important that whatever mode of treatment against fleas is chosen it should commence in late winter or early spring and continue throughout the summer months and into autumn. Traditionally this has entailed treatment with insecticides at monthly intervals throughout the active period of the fleas.

Application of the imidacloprid/flumethrin collars during late winter or spring will preclude the necessity of repeated treatments during the flea season. Collaring during late winter or spring will not only get rid of any existing flea population, but will also eliminate fleas that have over-wintered as pupae and have now accessed the cats in spring. Thereafter the collars will protect cats from re-infestation until the end of the flea season.

Equally important, residues of the imidacloprid component of the collars on bedding or in other resting places of treated cats will prevent any flea larvae that do hatch from developing into adults. Furthermore, flea control provided by the sustained efficacy of the imidacloprid/flumethrin collars will not only be apparent during the current flea season, but will also spill over into the following season because of the drastic reduction in the flea population in the first year of treatment.

The rapid effectiveness of the imidacloprid/flumethrin collars against an existing population of *C. felis felis* on cats, and its sustained almost immediate effect against re-infestation, coupled with the larvicidal efficacy of imidacloprid in the cat’s immediate surroundings, markedly reduces the chances of a cat developing flea allergy dermatitis. In addition *in vitro* experiments have demonstrated that cat hair, treated with minute amounts of imidacloprid, inhibits feeding by adult fleas [27].

The off-host lethal effect of imidacloprid against flea larvae should diminish the chances of larvae in the immediate surroundings of a collared cat ingesting the eggs of the cestode *D. caninum*. Even if fleas infected with the cysticercoids of *D. caninum* do get onto a collared cat, the rapidity with which the majority of them would be killed will conceivably also reduce the chances that the cat would swallow one while grooming. However, should infection with the tapeworm *D. caninum* be diagnosed in a cat, anthelmintic treatment is essential. Nevertheless, if this is not accompanied by a flea control program aimed at eliminating fleas on the host as well as flea larvae in its environment, long-term control of the cestode may not be achieved.

With the exception of some species, infestation with ticks in both Hemispheres mirrors that of fleas. It commences in spring or escalates from low numbers during spring to reach peak burdens in mid or late summer followed by a decline towards winter [26,28-31] The seasonal abundance of adult *R. turanicus* questing for hosts from the vegation also follows this pattern in South Africa [32].

Consequently treatment during this period is essential. However, unless the chemical chosen for treatment of ticks is also active against fleas, multiple treatments with more than one compound may be necessary. The combination of imidacloprid, which has excellent insecticidal properties, as shown in this and other studies, with flumethrin, with its excellent efficacy against many species of ticks, in a single slow-release matrix collar thus provides a sound alternative to a multi-chemical, multi-treatment regimen.

In the present studies the imidacloprid/flumetrin collars have proved to be highly effective against *C. felis felis* on cats, and the same level of efficacy can probably be expected against infestations with *C. canis* on these animals. The excellent efficacy of the collars demonstrated against *I. ricinus* in the laboratory studies will most likely also apply to *I. hexagonus* in the field in Europe and Great Britain, and probably to *Ixodes pilosus* in South Africa. The collars have also proved to be effective against infestations with lice, *Trichodectes canis*, and mites, *Sarcoptes scabiei* on dogs [33], and there seems to be no good reason to doubt that they will not be effective against the cat louse, *Felicola subrostratus*, and the cat scabies mite *Notoedres cati* on cats.

Other as yet speculative benefits are related to the prevention of flea or tick-borne pathogens. Several of these organisms require that the vector feeds for at least some period of time before transmission takes place [34]. The rapid elimination of both fleas and ticks by the active ingredients of the collars should certainly have an effect on disease transmission by these arthropods.

### Conclusion

The rapid insecticidal and acaricidal properties of the medicated collars against newly- acquired infestations of fleas and ticks and their sustained high levels of preventive efficacy have been clearly demonstrated. Taking into account the seasonality of fleas and ticks, the collars have the potential to prevent the transmission of vector-borne diseases and other conditions directly associated with infestation throughout the season of parasite abundance.

### Competing interests

These clinical studies were completely funded by Bayer Animal Health GmbH, Monheim, Germany, of which D. Stanneck (Germany) and K. Krieger are employees, and by Bayer HealthCare LLC, Animal Health (USA). ClinVet is an independent Contract Development Organisation, which was contracted to manage the conduct of a part of these studies. I.G. Horak is a long-term, contract employee of Clinvet and an Honorary Professor at the Universities of the Free State and Pretoria. All authors voluntarily publish this article and have no personal interest in these studies other than publishing the scientific findings that they have been involved in via planning, setting-up, monitoring and conducting the investigations and analysing the results.

### Author’s contributions

DS, EMK, JJF and WD designed the study design and protocols and JJF and EMK carried out the studies. DS, JJF, EMK and WD and IGH compiled and analysed the data. IGH was responsible for the first draft of the manuscript which was then substantially revised by all authors. All authors read and approved the final manuscript.

## References

[B1] Boreham RE Boreham PFL Dipylidium caninum: life cycle, epizootiology, and control Comp Con Educ 1990 12 667 675

[B2] Ménier K Beaucournu JC Importance médico-vétérinaire des puces du genre Ctenocephalides Stiles et Collins, 1930 Rev Méd Vét 1999 150 675 680

[B3] Otranto D Dantas-Torres F Canine and feline vector-borne diseases in Italy: current situation and perspectives Parasit Vectors 2010 3 2 10.1186/1756-3305-3-2 20145730 PMC2818618

[B4] Dryden MW Biology of fleas of dogs and cats Comp Con Educ 1993 15 569 578

[B5] Petney T Robbins R Robbins R Guglielmone A Panaskevich D Estrada-Peña A Horak I Shao R A look at the world of ticks Progr Parasit 2011 10.1007/978-3-642-21396-0_15

[B6] Ménier K Beaucournu JC Approche biogeographique du genre Ctenocephalides Stiles et Collins, 1930 (Insecta: Siphonaptera) Biographica 1999 75 79 88

[B7] Beaucournu JC Ménier K Le genre Ctenocephalides Stiles et Collins, 1930 (Siphonaptera, Pulicidae) Parasite 1998 5 3 16 9754292 10.1051/parasite/1998051003

[B8] Genchi C Traldi G Bianciardi P Efficacy of imidacloprid on dogs and cats with natural infestations of fleas, with special emphasis on flea hypersensitivity Vet Therap 2000 1 71 80 19757553

[B9] Koutinas AF Papazahariadou MG Rallis TS Tzivara NH Himonas CA Flea species from dogs and cats in northern Greece: environmental and clinical implications Vet Parasit 1995 58 109 115 10.1016/0304-4017(94)00706-I 7676591

[B10] Farkas R Gyurkovszky M Solymosi N Beugnet F Prevalence of flea infestation in dogs and cats in Hungary combined with a survey of owner awareness Med Vet Entomol 2009 23 187 194 10.1111/j.1365-2915.2009.00798.x 19712149

[B11] Supperer R Hinaidy HK Ein Beitrag zum Parasitenbefall der Hunde und Katzen in Ősterreich Deutsche tierärztliche Wochenschrift 1986 93 383 386 3536404

[B12] Chesney CJ Species of flea found on cats and dogs in south west England: further evidence of the polyxenous state and implications for flea control Vet Rec 1995 136 356 358 10.1136/vr.136.14.356 7610540

[B13] Xhaxhiu D Kusi I Rapti D Visser M Knaus M Lindner T Rehbein S Ectoparasites of dogs and cats in Albania Parasit Res 2009 105 1577 1587 10.1007/s00436-009-1591-x 19690887

[B14] Akucewich LH Philman K Clark A Gillespie J Kunkle G Nicklin CF Greiner EC Prevalence of ectoparasites in a population of feral cats from north central Florida during the summer Vet Parasit 2002 109 129 139 10.1016/S0304-4017(02)00205-4 12383632

[B15] Horak IG Beaucournu JC Braack LEO Parasites of domestic and wild animals in South Africa. XLIV. Fleas (Insecta: Siphonaptera: Pulicidae) collected from 15 carnivore species Onderstepoort J Vet Res 2004 71 9 14 15185570 10.4102/ojvr.v71i1.281

[B16] Ogden NH Cripps P Davison CC Owen G Parry JM Timms BJ Forbes AB The ixodid tick species attaching to domestic dogs and cats in Great Britain and Ireland Med Vet Entomol 2000 14 332 338 10.1046/j.1365-2915.2000.00244.x 11016442

[B17] Horak IG Heyne H Donkin EF Parasites of domestic and wild animals in South Africa. XLVIII. Ticks (Acari: Ixodoidea) infesting domestic cats and wild felids in southern Africa Onderstepoort J Vet Res 2010 77 Art. #3 7 10.4102/ojvr.v77i1.3 23327159

[B18] Walker JB Keirans JE Horak IG The genus Rhipicephalus (Acari, Ixodidae): a guide to the brown ticks of the World Cambridge 2000 Cambridge University Press, Cambridge

[B19] Jameson LJ Medlock JM Tick surveillance in Great Britain Vector-borne and Zoonotic Diseases 2011 11 10.1089/vbz.2010.0079 20849277

[B20] Horak IG Matthee S Parasites of domestic and wild animals in South Africa. XLIII. Ixodid ticks of domestic dogs and cats in the Western Cape Province Onderstepoort J Vet Res 2003 70 187 195 14621314

[B21] Schnieder T Wolken S Mencke N Comparative efficacy of imidacloprid, selectamin, fipronil-(S)-methoprene, and metaflumizone against cats experimentally infested with Ctenocephalides felis Vet Therap 2008 9 176 183 19003778

[B22] Hopkins TJ Kerwick C Gyr P Woodley I Efficacy of imidacloprid to remove and prevent Ctenocephalides felis infestations on dogs and cats Aust Vet Prac 1996 26 150 153

[B23] Jacobs DE Adulticidal and larvicidal effects of imidacloprid: two stage control Supplement Comp Con Educ Pract Vet 2000 22 15 17

[B24] Keil K Wellington J Ciszewski D Efficacy of Advantage® in controlling flea allergy dermatitis in cats Supplement Comp Con Educ Pract Vet 2002 24 6 9

[B25] Amin OM The fleas (Siphonaptera) of Egypt: distribution and seasonal dynamics of fleas infesting dogs in the Nile Valley and Delta J Med Entomol 1966 3 293 298 5986748 10.1093/jmedent/3.3-4.293

[B26] Horak IG Parasites of domestic and wild animals in South Africa. XIV. The seasonal prevalence of Rhipicephalus sanguineus and Ctenocephalides spp. on kennelled dogs in Pretoria North Onderstepoort J Vet Res 1982 49 63 68 7122067

[B27] Rust MK Hinkle NC Waggoner M Mencke N Hansen O Vaughn MB The influence of imidacloprid on adult cat flea feeding Comp Con Educ Pract Vet (Supplement) 2001 23 1 4

[B28] Földvári G Farkas R Ixodid tick species attaching to dogs in Hungary Vet Parasit 2005 129 125 131 10.1016/j.vetpar.2004.11.032 15817212

[B29] Smith FD Ballantyne R Morgan ER Wall R Prevalence, distribution and risk associated with tick infestation of dogs in Great Britain Med Vet Entomol 2011 10.1111/j.1365-2915.2011.00954.x 21418263

[B30] Koch HG Seasonal incidence and attachment sites of ticks (Acari: Ixodidae) on domestic dogs in southeastern Oklahoma and northwestern Arkansas, USA J Med Entomol 1982 19 293 298 7120309 10.1093/jmedent/19.3.293

[B31] Horak IG Jacot Guillarmod A Moolman LC De Vos V Parasites of domestic and wild animals in South Africa. XXII. Ixodid ticks on domestic dogs and on wild carnivores Onderstepoort J Vet Res 1987 54 573 580 3444612

[B32] Gallivan GJ Spickett AM Heyne H Spickett A Horak IG The dynamics of questing ticks collected for 164 consecutive months off the vegetation of two landscape zones in the Kruger National Park (1988 – 2002). III. The less commonly collected species Onderstepoort J Vet Res 2011 78 1 9 Art. #41 10.4102/ojvr.v78i1.41 23327206

[B33] Stanneck D Kruedewagen EM Fourie JJ Horak IG Davis W Krieger KJ Efficacy of an imidacloprid/flumethrin collar against fleas, ticks, mites and lice on dogs Parasit Vectorsin press 10.1186/1756-3305-5-102 PMC3433312 22647530

[B34] Coetzer JAW Tustin RC Infectious diseases of livestock 2004 Oxford University Press, Cape Town

